# The World Trade Center Residents’ Respiratory Health Study: New-Onset Respiratory Symptoms and Pulmonary Function

**DOI:** 10.1289/ehp.7375

**Published:** 2004-12-20

**Authors:** Joan Reibman, Shao Lin, Syni-An A. Hwang, Mridu Gulati, James A. Bowers, Linda Rogers, Kenneth I. Berger, Anne Hoerning, Marta Gomez, Edward F. Fitzgerald

**Affiliations:** ^1^New York University School of Medicine, Department of Medicine, Division of Pulmonary and Critical Care Medicine, New York, New York, USA; ^2^New York State Department of Health, Albany, New York, USA

**Keywords:** asthma, environmental disasters, environmental hazards, reactive airways dysfunction, World Trade Center

## Abstract

The destruction of the World Trade Center (WTC) on 11 September 2001 in New York City resulted in the massive release of pulverized dust and combustion products. The dust and smoke settled in the surrounding area, which encompassed a large residential community. We hypothesized that previously normal residents in the community surrounding the former WTC would have an increased incidence of persistent respiratory symptoms and abnormalities in screening spirometry. A hybrid cross-sectional and retrospective cohort study using a symptom-based questionnaire and onsite screening spirometry in residents in an exposed area and in a control area was performed 12 ± 4 months after the collapse. Surveys were analyzed from 2,812 residents. New-onset respiratory symptoms were described by 55.8% of residents in the exposed area, compared with 20.1% in the control area after the event. Persistent new-onset symptoms were identified in 26.4 versus 7.5% of residents in the exposed area versus control area, respectively. No differences in screening spirometry between the groups were detected. A small pilot study suggested the possibility of an increase in bronchial hyperresponsiveness in exposed participants with persistent symptoms. The data demonstrate an increased rate of new-onset and persistent respiratory health effects in residents near the former WTC compared with a control population.

The destruction of the World Trade Center (WTC) in New York City on 11 September 2001 resulted in the pulverization of two 107-story buildings and the massive release of combustion products from jet fuel and burning structures. An initial cloud of dust and smoke enveloped the area in all directions. Subsequent wind-blown plumes dispersed dust and smoke throughout lower Manhattan and Brooklyn. Fires in the 16-acre site continued for more than 3 months after the event, with the prolonged release of combustion products. Analyses of the settled dusts have revealed cement, glass, and particulate matter, including gypsum, calcium carbonate, cement dust, and glass fibers. The dusts were alkaline, with a pH ranging from 9.3 to 11.5 (Lioy et al. 2002; McGee et al. 2003; Service 2003). Metals, including chromium, iron, magnesium, manganese, aluminum, barium, titanium, and lead, were also detected (Lioy et al. 2002). Particles were also noted to contain polycyclic aromatic hydrocarbons, polychlorinated biphenyls, and organochlorine pesticides (Lioy et al. 2002; Offenberg et al. 2003).

Although often considered a financial district, lower Manhattan contains a large residential community with approximately 58,000 residents living south of Canal Street. The residential communities encompass many socioeconomic levels and residents of diverse race/ethnicity. Housing stock consists of large housing complexes containing thousands of residential units as well as smaller residential buildings. Some residents in the immediate area surrounding the former WTC [Ground Zero (GZ)] were immediately evacuated; however, many remained in their apartments. Residents who were evacuated returned to their apartments over the ensuing weeks to months. Dusts from the collapse settled on streets, playgrounds, cars, and buildings. Dusts entered apartments through open windows, building cracks, and ventilation systems. Removal of these dusts in individual apartments was accomplished in a variety of ways; some residents used professional cleaners, whereas many performed the operation themselves. No consistent cleanup operation was offered to the residential community until 1 year after the event.

Adverse respiratory health effects from exposures to WTC dusts are being reported. Firefighters exposed to materials generated during the collapse of the WTC have developed cough and bronchial hyperresponsiveness (Banauch et al. 2003; Prezant et al. 2002). A preliminary telephone survey of a small sample of residents in Manhattan also suggested the presence of respiratory health effects 8 weeks after the event (Fagan et al. 2002). To examine whether the destruction of the WTC resulted in adverse respiratory health effects in the residential community, we developed a collaborative effort between the New York State Department of Health, New York University School of Medicine/Bellevue Hospital, and numerous community health programs and local community organizations. The overall study was designed to test the hypotheses that the rates and severity of new and previously existing respiratory diseases increased among residents after 11 September 2001 in the community surrounding GZ compared with a control community. We now report results of the first part of the study, which was designed to test the hypothesis that the destruction of the WTC increased the incidence of persistent new-onset respiratory symptoms and airflow obstruction in previously normal residents in the surrounding community. Additional studies will address upper respiratory symptoms, exacerbations of preexisting asthma, and medical care utilization.

## Materials and Methods

### Study participants.

Because of the unforeseen nature of the event, the study was necessarily designed as a hybrid cross-sectional and retrospective cohort study of residents in an exposed area and a control area and was approved by institutional review boards of the New York State Department of Health and New York University. All participants gave their written consent. Community residents, advocacy groups, local community boards, local tenant organizations, local medical organizations, and clinics all actively participated in the design and implementation of the study. Residents in buildings within a 1-mile radius from the former WTC were considered to be in the exposed area. Building complexes in the exposed area were identified in each direction of GZ. Residents in buildings located > 4.8 miles north of the WTC in Manhattan were considered to live in the control area. Areas south, east, and west of the WTC that were affected by the plume were excluded from selection as a control area. Building complexes in both the exposed area and the control area were identified by type of housing unit (e.g., low- or high-income rental, cooperative, condominium, or federally funded housing complex) to obtain a distribution of socioeconomic levels in the survey. Building complexes with similar characteristics were identified for the exposed area and the control area. Residual socioeconomic differences among the study areas were controlled for during analysis. We oversampled the population in the exposed area to obtain a large and representative population. In addition, at the time that this study was developed and implemented, this was the only study of health effects of local residents, and we thought that the detection of individuals in this study might provide the only opportunity for identification of residents for follow-up studies of health effects. We used the ratio of 9:1 (exposed:control area) while recruiting study participants. The exposed area included 49 buildings in lower Manhattan composed of approximately 9,200 households. The control area included approximately 1,000 households.

### Study procedure.

A self-administered questionnaire to identify asthma or asthmalike symptoms was developed from previously validated questionnaires (Abramson et al. 1991; Asher et al. 1995; Burney et al. 1989; Ravault and Kauffmann 2001). Questions were modified to delineate reference times in relation to 11 September 2001, to identify the onset of symptoms, and to determine whether symptoms were present within the 4-week period of responding to the questionnaire. Additional questions were included to obtain demographic information as well as to identify the presence of the resident in the apartment during the time of interest and any preceding or subsequent medical problems and medications. Questionnaires were available in Spanish and traditional Chinese.

The study was publicized at community board meetings, tenant meetings, local health fairs, building luncheons, and meetings. Notices of the study were included in local newspapers and building newsletters. Postings were also placed in buildings and streets. Outreach workers were situated in buildings at the time of delivery of the questionnaires to help distribute questionnaires and respond to questions.

Questionnaires were distributed to all defined buildings 12 ± 4 months after the collapse of the WTC. Questionnaires were initially distributed via bulk mail. However, it became apparent that the federal postal service was not functioning in many of the areas near GZ in a consistent manner and that many of the questionnaires had not been delivered. Subsequently, in areas with questionable mail delivery, questionnaires were hand delivered to every apartment or, when entry was denied, to every building lobby. A first-class mailing of the questionnaire was then repeated, and all apartments were sent reminder postcards. Up to four residents (two adults, two oldest children) in each apartment were asked to complete the questionnaire.

Because of a concern about potential selection bias in our response, two buildings of similar housing stock were targeted in the exposed area and in the control area for more intensive outreach. Residents of these buildings received a third copy of the packets, and outreach workers remained in these buildings for additional days and evenings to respond to questions and reinforce participation in the study. These targeted buildings represented 440 households in the exposed area and 240 in the control area. These targeted buildings with higher response rates were used to provide an estimate of selection bias compared with the remaining study sites.

### Case definitions.

“Previously normal” residents were considered to be those who did not have a physician diagnosis of asthma, chronic obstructive pulmonary disease, or emphysema before 11 September 2001.

Previously normal residents with new-onset symptoms were considered those who answered positively to any of the questions pertaining to respiratory symptoms of cough, shortness of breath (SOB), or wheeze or were using oral or inhaled medications for asthma at any time after 11 September 2001. Previously normal residents with persistent new-onset symptoms were defined as participants with symptoms that began after 11 September 2001 and who had a frequency of symptoms more than twice each week or medication use within 4 weeks of responding to the questionnaire.

### Screening spirometry.

Participants who were previously normal and had persistent new-onset symptoms were invited to perform a scheduled screening spirometry at a local community site. Participants were excluded for analysis if they were < 6 years of age because of the potential for technical difficulties. Participants > 65 years of age or with a history of cardiovascular disease were excluded for safety reasons, because the studies were performed in the field. Participants with a current or > 5 pack-year history of cigarette use, who lived in the control area but worked in the exposed area, who returned to the residence after January 2002, or who refused to be recontacted were also excluded.

Spirometry was performed in the field by trained personnel with a Micro Direct (Lewiston, ME) portable spirometer that complied with American Thoracic Society specifications. Studies with three measurements within 5% of each other were considered acceptable. Participants on medications were asked to withhold use of medications for at least 4 hr. Values of forced expiratory volume in 1 sec (FEV_1_), forced vital capacity (FVC), FEV_1_/FVC, and flows at mid lung volumes (FEF_25–75_) were obtained. Analyses were performed using normal predicted values from Hankinson et al. (1999). Because studies were being performed in the community, bronchodilator responses were not assessed.

### Airway hyperresponsiveness.

Previously normal participants were invited to perform a methacholine challenge test (MCT) as a monitor of bronchial hyperresponsiveness. Participants < 55 years of age with an FEV_1_ ≥70% predicted and either persistent new-onset symptoms or absence of symptoms were invited to undergo an MCT at the New York University/Bellevue Hospital pulmonary function laboratory. Spirometry was performed to confirm baseline values. MCT was performed using the 2-min tidal breathing protocol with methacholine delivered via a nebulizer up to a maximal dose of 8 mg/mL (Crapo et al. 2000). A test was considered positive if the subject had a ≥20% drop in FEV_1_.

### Statistical methods.

We calculated the overall response rate on the basis of the number of households responding in the exposed area and control area because of the variation in the number of individuals residing in each household. An undetermined number of residents permanently moved out of the exposed area after the event. Packets that were returned unopened were therefore considered to have come from vacant households and were considered vacant for this calculation. The rates for each health outcome were calculated as the number of participants with a specific outcome, divided by the number of eligible participants. We computed cumulative incidence ratios (IRs) comparing the exposed area and control area and used 95% confidence intervals (CIs) to estimate the precision of the cumulative IR. We used unconditional logistic regression analysis to compute adjusted odds ratios (ORs) while controlling for potential confounders, including age, sex, education, race, and smoking. Because respiratory diseases are not rare events, ORs from logistic regression tended to persistently overestimate cumulative IRs. Therefore, the crude IRs with 95% CI are presented in result tables, and adjusted OR as well as CIs were used only to examine if the results were still statistically significant after controlling for confounders.

The demographic characteristics between the participants in the exposed area and control areas were compared using the *t*-test of continuous variables (e.g., age) or the chi-square test for categorical variables (e.g., sex). For the analysis of the spirometry data, means ± SDs are presented. The *t*-test was used to compare the mean in the exposed area with the mean in the control area.

## Results

### Study participants.

A total of 9,168 survey packages were sent to households in the exposed area and 962 to households in the control area. Responses were obtained from 2,520 households in the exposed area (22.3%) and 295 in the control area (23.3%). Household responses were greater in the targeted buildings, with 205 of 440 households responding from the exposed area (43.8%) and 99 of 240 (41.2%) households responding from the control area.

**Figure 1:**
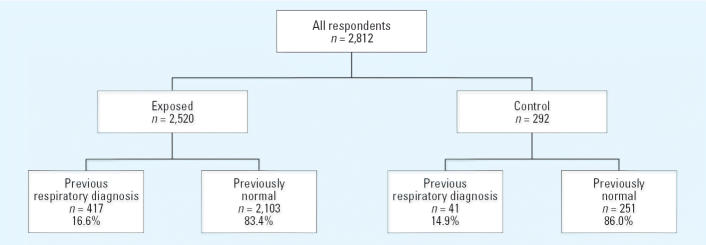
Study cohort classifications. Previously normal residents were considered to be those who did not have a physician diagnosis of asthma, chronic obstructive pulmonary disease, or emphysema before 11 September 2001.

A total of 3,196 individual responses were returned for analysis; 384 respondents were excluded from analysis because they did not reside in the residence on 11 September 2001, they returned to the residence after 1 January 2002, the residence was in the control area but the respondent worked in the exposed area, or the questionnaire was answered for a person born after 11 September 2001. Of the 2,812 responses that were therefore used for analyses, 2,520 were returned from residents in the exposed area, and 292 from residents in the control area (see Figure 1).

The demographic characteristics of the 2,812 remaining respondents are shown in Table 1. In both the exposed area and the control area, there were more women respondents than men respondents. Although most respondents were between 35 and 64 years of age, there was a higher response rate from older participants in the control area. A wide distribution of income levels was detected in both the exposed area and control area; however, more respondents from the exposed area earned < $25,000 compared with those in the control area. More respondents in the control area were Caucasian, whereas more respondents in the exposed area were of Asian or Hispanic/Latino descent. These differences reflect differences in the underlying populations according to the 2000 U.S. Census (U.S. Census Bureau 2000) and were considered potential confounders. As such, they were controlled for in multivariate analyses.

### Respiratory symptoms in residents.

A previous diagnosis of respiratory disease was identified in 417 (16.6%) and 41 (13.9%) of the residents in the exposed area and the control area, respectively. These residents were not considered previously normal and were excluded from subsequent analysis. Thus, information from 2,103 participants in the exposed area and 251 participants in the control area was available for analysis.

As shown in Table 2, more than twice as many previously normal residents in the exposed area complained of respiratory symptoms at some time after the collapse of the WTC compared with residents in the control area. Cough was the most common symptom and was noted in three times as many participants in the exposed area as in the control area. Four times as many residents in the exposed area complained of wheeze compared with residents in the control area. Approximately three times as many residents in the exposed area complained of SOB. The difference in these symptoms in the residents in the exposed area remained significant even after adjusting for age, sex, education, smoking, and race.

To assess whether respiratory symptoms were persistent, previously normal participants were asked about the presence and frequency of individual symptoms within the 4 weeks preceding the survey. Symptoms were considered persistent if they occurred with a frequency of at least twice each week. As shown in Table 3, symptoms had resolved in many of the residents by the 4 weeks preceding the survey. However, almost three times the number of residents in the exposed area continued to have any persistent respiratory symptom compared with residents in the control area. The predominant symptom remained cough. Persistent wheezing was reported in 10.5% of participants in the exposed area compared with 1.6% in the control area.

Similar results were noted for the targeted population that received intensive outreach and had a greater response rate (43.8 and 41.2% response rate for exposed area and control area, respectively) compared with the total study population. Respondents from the targeted exposed area had a greater risk of new-onset respiratory symptom (IR, 3.05; 95% CI, 2.12–4.39) and persistent respiratory symptoms (IR, 4.63; 95% CI, 2.50–8.57) compared with residents in the targeted control area. Persistent daytime SOB was reported in 13.7%, and wheezing was reported in 13.7% of these previously normal residents.

We assessed the severity of the reported persistent symptoms, as defined by the frequency of each individual symptom, in previously normal participants with persistent new-onset symptoms. This analysis is shown in Table 4. Almost 24% of participants with a persistent symptom complained of cough on a daily basis. Daily wheezing was described by 17.5% of the residents in the exposed area who had a persistent symptom. Using frequency of symptoms to characterize severity of asthma according to the Global Initiative for Asthma guidelines (National Heart, Lung, and Blood Institute 2002), this symptom frequency would be compatible with at least moderate persistent asthma.

### Screening spirometry in residents.

Three hundred sixteen participants were eligible and agreed to screening spirometry in the field. Many residents did not respond to repeated attempts at telephone scheduling, failed to come to the scheduled appointments, or could not complete a successful study. Spirometry was successfully completed in 117 (37%) of the eligible residents. No differences were detected between residents with symptoms in the exposed area compared with asymptomatic residents in any parameter of airflow, including FEV_1_, FVC, FEV_1_/FVC, and FEF_25–75_ (Table 5). We failed to observe a difference in the number of individuals with an FEV_1_ or FEV_1_/FVC below the lower limit of normal in the individuals in the exposed area with new-onset persistent symptoms and asymptomatic individuals, or between individuals in the control area. Of participants with persistent symptoms in the exposed area, 20.8% had used a controller medication (inhaled corticosteroid, long-acting β-agonist, theophylline compound, leukotriene modifier) in the 4 weeks before spirometry, compared with none of the participants in the asymptomatic groups. Of participants with persistent symptoms in the exposed area, 16.7% had used a short-acting β-adrenergic agonist inhaler for asthma, compared with 1.5% in the asymptomatic exposed group and none in the control residents.

All participants were invited to undergo an MCT according to eligibility criteria defined in “Materials and Methods.” MCT was performed in 24 volunteer participants, including those with persistent new-onset respiratory symptoms (*n* = 12), asymptomatic participants from the exposed area (*n* = 6), and asymptomatic participants from outside the exposed area (*n* = 6). No significant difference was noted in baseline spirometry between these groups (data not shown). Many (6 of 12) participants with persistent new-onset symptoms had a positive MCT compared with asymptomatic participants (*p* < 0.05). None of the asymptomatic participants in either group had a positive MCT.

## Discussion

The World Trade Center Residents’ Respiratory Health Study was initiated in response to questions by residents in the surrounding community of the disaster site about the respiratory health risk for residents and was designed to study upper and lower respiratory tract symptoms, physician diagnoses, unplanned medical visits, and physical condition of the apartments after the collapse of the WTC. We now report on the presence and persistence of new respiratory health issues in residents near GZ. The study was completed 16 months after the destruction of the WTC. Our study suggested an increased incident rate of new-onset respiratory symptoms in residents near GZ compared with residents in a control area. Although these symptoms resolved in many residents, an increased incident rate of persistent new-onset respiratory symptoms was also detected compared with a control group. These data suggest that exposure to dust and fumes from the destruction of the WTC was associated with new-onset respiratory symptoms that persisted in a subset of residents.

The predominant respiratory symptom detected in symptomatic residents consisted of cough, with some participants also experiencing dyspnea and wheezing. These symptoms are consistent with those identified in the rescue workers and responder populations such as the firefighters and ironworkers (Feldman et al. 2004). They fit some but not all criteria for reactive airways dysfunction (RADS) (Alberts and do Pico 1996; Bardana 1999; Brooks et al. 1985). We cannot document the exposure level of the residents to the dusts and fumes, and although some of these residents may have had high-level exposure from the initial dust cloud, others may have only experienced lower-level exposure from settled dust and persistent fires. Descriptions of irritant-induced asthma have included cases with a history of repeated low-intensity exposures, in which the symptoms have a more delayed expression, and this pattern may be more consistent with the potential exposure history and symptoms of many of the residents in this study (Brooks 1998; Kipen et al. 1994). The persistence of symptoms identified in some of the study participants is also consistent with irritant-induced asthma, in which symptoms can persist for years (Chang-Yeung et al. 1994; Demeter et al. 2001). The persistence of symptoms is also consistent with the findings recently described in firefighters exposed to WTC dusts (Banauch et al. 2003).

Only a subset of residents with potential exposure experienced the onset and persistence of respiratory symptoms. The characteristics of this susceptible group are unclear. The variation in response may be due to differences in the intensity or duration of exposure to the WTC dusts in the population with persistent symptoms compared with those without. Alternatively, irritant-induced asthma has been described to be more common in participants with preexisting allergic or atopic disorders (Brooks et al. 1998). We did not specifically explore whether participants with persistent symptoms had preexisting atopic disorders in this study.

The possibility exists that psychological stress might play a role in the reported symptoms, because post-traumatic stress disorder has been reported to be associated with asthma and other respiratory diseases (Fagan et al. 2003). In the present study we could not determine whether environmental factors, psychological distress, or a combination, contributed to the increase of respiratory symptoms, because psychological factors were not examined in this part of the study.

Despite our original hypothesis, we were unable to detect a significant difference in airflow parameters measured by screening spirometry performed in the field between residents with persistent new-onset respiratory symptoms and asymptomatic or control residents. We did not have preexisting medical information available to us for the population of study, and as a result, we performed between-subject comparisons. The possibility exists that our statistical power was not great enough to detect small differences in airflow measurements between the two populations. The symptoms detected in the exposed population may also be due to changes in the small airways or to an increase in bronchial hyperresponsiveness, both of which can be missed with routine screening spirometry. In addition, many participants in the group with persistent symptoms in the exposed area were using a controller medication at the time of the study. Use of these medications may have improved their lung function. The findings are, however, consistent with those described in firefighters exposed to WTC dusts in which no significant differences in spirometry values were detected between participants with high and low exposure, suggesting that these parameters are insensitive for between-subject comparisons in these exposed populations (Prezant et al. 2002). MCT performed in a small pilot study of participants suggested that the symptoms of some of these residents might be explained by the presence of bronchial hyperresponsiveness, a finding that would be consistent with the data reported for firefighters (Prezant et al. 2002).

The predominant compounds detected in the settled dusts collected 1 and 2 days after the WTC explosion included calcium sulfate (gypsum) and calcium carbonate (calcite) (Lioy et al. 2002; McGee et al. 2003; Service 2003). The aqueous extracts were extremely alkaline (Lioy et al. 2002). These particle characteristics are associated with mucus membrane irritation and thus have the potential to elicit airway symptoms consistent with those detected in this study (Stellman 1998). Biologic plausibility for health effects from WTC dusts is supported by *in vitro* and *in vitro* studies. Primary human lung cells (alveolar macrophages and epithelial cells) reveal an increase in inflammatory cytokines, interleukins 8 and 6, in response to WTC dusts (Payne et al. 2004). Animal studies, using WTC-derived fine particulate matter, demonstrate that very high doses elicit pulmonary inflammation and hyperresponsiveness (Gavett et al. 2003). Although lower doses of these particles did not induce inflammation or hyperresponsiveness, the effects of chronic exposures were not tested in these studies.

Despite the large sample size of this study, there are some potential limitations to the study. In contrast to the firefighters, in whom a baseline health and pulmonary function profile was well established and documented before 11 September 2001, no consistent information was available about the health of the residents in the surrounding GZ community before 11 September 2001. Many of these residents were considered normal before that date and thus do not have documented respiratory health information preceding 11 September 2001. We therefore used self-reported health information. The possibility of reporting bias or differential recall by persons in the different study areas exists. To minimize this possibility, questions about health problems that should be unrelated to WTC events were also included in the questionnaire. The similar rate of problems such as disability affecting physical activity in the two areas (14.2 and 13%, respectively) suggested the absence of significant reporting bias due to residence area. Participants responding affirmatively about every symptom may have been affected by recall bias. Ten of the respondents answered in this way; however, minimal changes were observed when these individuals were excluded from the analysis. We also obtained information about unplanned medical visits in the months after the WTC collapse, events that may be more memorable than symptoms. Unplanned medical visits for respiratory problems were significantly increased in the affected area (14.7%) over the control area (8.4%) [cumulative incidence ratio (CIR), 1.76; 95% CI, 1.15–2.68)] after controlling for potential confounders. A significantly higher proportion of affected area residents started using respiratory medication after 11 September 2001 (17.9%) compared with controls (6.2%) (CIR, 2.88; 95% CI, 1.75–4.75). We also compared the proportion of respondents reporting a specific respiratory symptom and unplanned medical visits in both areas. We found that the proportions were similar in the affected and control areas for most symptoms. If there had been overreporting in the affected area, the proportion of individuals reporting a specific symptom who also had unplanned medical visits should have been lower in the affected area than in the control area.

Despite the active involvement of the community in the design and implementation of this study, the response rate in both the exposed area and control area was low. Several possible explanations can be suggested for this low response rate. First, although we used many means to deliver the questionnaires, the absence of reliable mail in many of the exposed areas may have reduced our ability to reliably distribute the surveys. Moreover, because of the well-documented emotional aftermath of the event, many residents may have been unwilling to answer questions that may have provoked sensitive emotions even 1 year after the event. In addition, at the time of the study, residents were also receiving forms from many other agencies. Both confusion over which studies were being completed, and study fatigue may have occurred. Finally, we were unable to determine a true response rate because a significant number of residents permanently moved out of the exposed area after 11 September 2001. In some buildings, residents estimated that > 50% of the occupants had moved from the buildings. We were unable to obtain a listing of residents in the area before and after the event, and for this reason, the denominator for calculating the household response rate may have been an overestimate, resulting in an underestimate of the actual response rate. Furthermore, low response rates are common for studies performed in New York City; the 2000 Census recorded only a final response rate in New York City of 55% despite intense advertising and door-to-door follow-up (U.S. Census Bureau 2000).

The potential for selection bias exists in this self-administered survey, and it is possible that residents with new-onset respiratory symptoms may have been more likely to participate in this study compared with those without symptoms. Several procedures were used during the study in an attempt to minimize this potential problem. The importance of participation for residents with and without breathing problems was stressed in all announcements of the study. In addition, a target population that received intensive outreach was studied in both the exposed area and the control area. This target population, which had a higher response rate compared with the study population as a whole, demonstrated an even greater increase in persistent symptoms in residents in the exposed area compared with the control area, with an increase in individual symptoms ranging from 14 to 63% in the target population. Had there been a significant selection bias or an overestimation of the association, analysis of the target population should have demonstrated a weaker exposure–disease association compared with the control population. In contrast, analysis of symptoms in the target population demonstrated that increases in new-onset symptoms were consistently and significantly higher in the exposed areas compared with the control area. This finding suggests that if there were selection bias, it would be in the opposite direction (i.e., the true association would be underestimated).

A plume dispersion model is not yet complete by the U.S. Environmental Protection Agency and thus was not available to us to allow a detailed exposure assessment. However, we obtained self-report information on the condition of the individual households as a possible surrogate for exposure. Many of the apartments that were in close proximity to GZ were severely damaged by the event. Apartments that surrounded GZ in all directions were covered in dusts from the initial dispersion. The presence of persistent new-onset respiratory symptoms was significantly associated with the presence of physical damage of the apartment, dust on the surfaces, or a long duration of dust or odors (data not shown). In addition, residents who were south of Canal Street in lower Manhattan on 11 September 2001 (i.e., in close proximity to the WTC) were at higher risk of developing persistent new-onset respiratory symptoms compared with residents who were not in the area on the day of the event.

The possibility of exposure misclassification may also exist. To minimize this bias, we excluded individuals who had moved out of their residence for a prolonged period of time or who may have had exposure that was unrelated to their area of residence. Some residents may have altered their behavior and spent less time at home in the aftermath of 11 September 2001; however, we would not be able to identify these residents. In addition, because of wind, it is also possible that the WTC dust plume also affected residents in the control area.

## Conclusion

These data suggest that residents living in the community surrounding the former WTC experienced a higher rate of adverse respiratory health effects 1 year after the event compared with a control population. Respiratory symptoms consisted of cough, dyspnea, and wheeze. Although most of these symptoms resolved by approximately 12 months after the event, a significant number of residents continued to have persistent new-onset respiratory symptoms. Abnormalities in screening spirometry failed to explain the symptoms in these participants, and additional tests, including tests for bronchial hyperresponsiveness, may be helpful to further characterize these symptoms. Biologic plausibility for these complaints is provided by chemical analysis of the settled dusts and animal studies. Long-term health effects remain unknown and warrant further investigation and follow-up of exposed residents.

## Figures and Tables

**Table 1:** Demographics of resident respondents (percentage) to the WTC Residents’ Respiratory Health Study.

Characteristic	Exposed (*n* = 2,520)	Control (*n* = 292)	*p*-Value
Sex			
Male/female	38.0/62.0	41.3/58.7	0.3056
Age (years)			
0–34	23.4	23.8	
35–64	51.7	35.9	< 0.0001
≥65	24.9	40.3	
Household income			
< $24,999	33.4	20.3	
$25,000–49,999	18.3	19.8	0.0004
$50,000–99,999	23.6	30.4	
≥$100,000	24.6	29.5	
Race/ethnicity*^a^*			
White	61.7	79.3	< 0.0001
Hispanic	13.7	7.5	0.0038
Asian	16.4	3.2	< 0.0001
African American	8.2	11.8	0.0439
Other	4.5	4.4	0.9515
Education			
< High school	19.6	10.8	0.0004

a Race/ethnicity groups are not mutually exclusive and therefore do not add to 100%.

**Table 2:** New-onset respiratory symptoms (percentage) in previously normal residents.*^a^*

Symptom	Exposed (*n* = 2,103)	Control (*n* = 254)	Crude IR (95% CI)
Any cough without cold	40.6	12.1	3.36 (2.38–4.74)*
Nighttime cough	36.7	11.7	3.15 (2.21–4.48)*
Wheeze	28.4	6.6	4.32 (2.68–6.98)*
Daytime SOB	27.2	10.4	2.62 (1.80–3.83)*
Morning chest tightness	23.7	7.9	3.00 (2.15–6.94)*
SOB after exercise	18.1	4.7	3.86 (2.15–6.94)*
Nighttime SOB	15.8	4.5	3.48 (1.94–6.25)*
Any of the above symptoms	55.8	20.1	2.78 (2.17–3.56)*

a No diagnosis of asthma, chronic obstructive pulmonary disease, chronic bronchitis, or other lung disease before 11 September 2001.

*Effect still statistically significant after adjusting for age, sex, education, smoking, and race.

**Table 3:** Persistent*^a^* new-onset respiratory symptoms (percentage) in previously normal residents.

Symptom	Exposed (*n* = 2,103)	Control (*n* = 254)	Crude IR (95% CI)
Cough without cold	16.0	4.0	3.99 (2.15–7.38)*
Nighttime cough	12.9	3.7	3.51 (1.83–6.72)*
Wheeze	10.5	1.6	6.50 (2.44–17.33)
Daytime SOB	10.6	3.6	2.94 (1.53–5.66)*
Morning chest tightness	8.4	1.6	5.21 (1.95–13.91)*
SOB after exercise	7.4	1.7	4.45 (1.66–11.91)*
Nighttime SOB	6.2	0.8	7.64 (1.90–30.70)*
Any of the above symptoms	26.4	7.5	3.53 (2.28–5.47)*

a Symptom frequency ≥2 days per week in the past 4 weeks.

*Effect still statistically significant after adjusting for age, sex, education, smoking, and race.

**Table 4:** Frequency (percentage) of specific symptoms in residents with persistent new-onset respiratory symptoms.

Symptom	Never	< 2 days/week	2–6 days/week	Daily
Cough without cold	15.2	21.4	40.0	23.5
Nighttime cough	24.3	23.5	36.2	15.9
Daytime SOB	28.7	25.3	28.2	17.8
Wheeze	34.7	22.3	25.5	17.5
Morning chest tightness	41.9	20.4	27.5	10.2
SOB after exercise	44.1	19.7	24.3	11.8
Nighttime SOB	52.3	20.6	19.2	7.9

**Table 5:** Screening spirometry in previously normal residents with persistent new-onset respiratory symptoms (mean ± SD).

	Exposed persistent symptoms (*n* = 49)	Exposed asymptomatic (*n* = 67)	Control asymptomatic (*n* = 17)
FEV_1_ (percent predicted)	91.4 ± 12.1	95.4 ± 14.0	93.0 ± 11.9
FVC (percent predicted)	89.6 ± 12.2	94.3 ± 14.7	89.9 ± 10.0
FEV_1_/FVC (%)	82.1 ± 6.9	81.4 ± 4.2	83.3 ± 7.1
FEF_25–75_ (percent predicted)	90.1 ± 26.6	90.0 ± 22.0	94.6 ± 34.3
PEF (percent predicted)	90.2 ± 17.6	97.5 ± 15.9	93.8 ± 13.4

PEF, peak expiratory flow. *p*-Value > 0.05 for all comparisons.
